# Selection of Reliable Reference Genes for Analysis of Gene Expression in Spinal Cord during Rat Postnatal Development and after Injury

**DOI:** 10.3390/brainsci10010006

**Published:** 2019-12-20

**Authors:** Ján Košuth, Martina Farkašovská, Filip Mochnacký, Zuzana Daxnerová, Juraj Ševc

**Affiliations:** Institute of Biology and Ecology, Faculty of Science, P.J. Šafárik University in Košice, Šrobárova 2, 04154 Košice, Slovakia; jan.kosuth@upjs.sk (J.K.); martina.farkasovska@student.upjs.sk (M.F.); filip.mochnacky@student.upjs.sk (F.M.); zuzana.daxnerova@upjs.sk (Z.D.)

**Keywords:** reference gene, normalization, RT-qPCR, spinal cord injury, postnatal development, spinal cord

## Abstract

In order to obtain unbiased results of target gene expression, selection of the most appropriate reference gene (RG) remains a key precondition. However, an experimental study focused on the validation of stably expressed RGs in the rat spinal cord (SC) during development or after spinal cord injury (SCI) is missing. In our study, we tested the stability of the expression of nine selected RGs in rat SC tissue during normal development (postnatal days 1–43, adulthood) and after minimal (mSCI) and contusion (cSCI) spinal cord injury. The following RGs were tested: common housekeeping genes of basal cell metabolism (*Gapdh*, *Hprt1*, *Mapk6*) and protein translation (*Rpl29*, *Eef1a1*, *Eif2b2*), as well as newly designed RGs (*Gpatch1*, *Gorasp1*, *Cds2*) selected according to the RefGenes tool of GeneVestigator. The stability of RGs was assessed by geNorm, NormFinder, and BestKeeper. All three applets favored *Gapdh* and *Eef1a1* as the most stable genes in SC during development. In both models of SCI, *Eif2b2* displayed the highest stability of expression, followed by *Gapdh* and *Gorasp1*/*Hprt1* in cSCI, and *Gapdh* and *Eef1a1* in the mSCI experiments. To verify our results, selected RGs were employed for normalization of the expression of genes with a clear biological context in the SC—*Gfap* and *Slc1a3*/*Glast* during postnatal development and *Aif1*/*Iba1* and *Cd68*/*Ed1* after SCI.

## 1. Introduction

Myriads of processes at cellular and subcellular levels shape the developing parts of the nervous system into their final complexion. One can assume that an understanding of the various pathological conditions affecting the adult nervous system is inseparably linked with an understanding of its development and maturation.

These developmental or pathological processes could be studied on various levels by methods of molecular biology, microscopy, electrophysiology, etc. At the level of gene expression, there are several methods designated to reveal the presence and quantity of mRNA in a biological sample at a given moment, such as quantitative real-time RT-PCR (RT-qPCR), in situ hybridization, DNA microarrays, or RNA-seq. In spite of the progress and innovations in the abovementioned methods, RT-qPCR remains the method of choice for routine quantification of gene expression levels in biomedical research [1]. This is mainly due to its accessibility; the reasonable combination of easy feasibility, high sensitivity, and resolution; and the possibility to analyze and compare the expression of a single gene/relatively small set of genes in multiple samples. On the other side, the accuracy of the quantification using RT-qPCR relies on proper normalization, i.e., elimination of the non-biological variations caused by differences in the quantity and integrity of the RNA template, RNA recovery, and efficiency of cDNA synthesis [2].

The most preferred approach for the normalization of target gene (TG) expression in the sample is the application of an endogenous reference gene (RG), which serves as an internal control. The ideal RG should to be stable (in terms of expression) across the tested experimental conditions, such as development/differentiation, experimental treatments, or external stimuli. This is especially important when the expression of TG is examined during pathological conditions or development. Historically, the “housekeeping genes” encoding for cellular maintenance proteins were initially believed to be ideal RGs that are ubiquitously expressed and whose mRNA was thought to have general, uniform, and unchanging expression in different tissues and under different conditions [3,4]. Unfortunately, this idea remains unfulfilled, since the expression of all genes changes in time or is up- or downregulated under different conditions. This is even true for presumably stable housekeeping genes [5,6].

Therefore, in order to obtain unbiased results of target gene expression in routine experimental studies, the selection of the most appropriate (the most stably expressed) reference gene remains the key precondition. Nevertheless, the issue of validity of the reference gene in given experimental conditions is often underrated and thus the RGs may also be affected by the experiment [7,8], resulting in biased normalization of TG expression.

Indeed, studies of gene expression in the spinal cord persistently neglect RGs’ stability testing. The majority of studies dealing with gene expression during postnatal development and after spinal cord injury circumvent RG validation. Thus, the authors often rely on the routinely used housekeeping genes, mostly e.g., *Gapdh* [9,10], *β-actin* [11,12], or 18S rRNA [13,14], without testing and validating its stable expression under particular experimental conditions. To our knowledge, we are not aware of a serious survey and validation of stably expressed RGs in the rat spinal cord across postnatal development or after SCI. The issue was partially addressed by the study of Sundaram et al. [15], who focused on the appropriate use of methods validating RGs’ stability in the murine spinal cord. Several other papers deal with the validation of stable RGs in the spinal cord by neuropatic pain after spared nerve injury (SNI) and peripheral nerve injury (PNI) or after inflammatory injury [16,17,18]. The stability of RGs’ expression is assessed by several mathematical models based on rating a panel of preselected RGs according to their variability and inter-/intra-genic correlations. The most frequently used applets evaluating the variability are geNorm [19], BestKeeper [20], and NormFinder [21]. Besides the common housekeeping genes, the candidate RGs assessed for the actual experiment can be preselected by the “RefGenes” tool of GeneVestigator^®^ [5]. The platform facilitates the search for stably expressed genes in the microarray and RNA-seq data of the fully curated GeneVestigator^®^ database.

The objective of our work was to identify a proper reference gene applicable for the normalization of gene expression in the rat spinal cord in developmental studies and in studies exploiting SCI. In our study, we tested the stability of nine selected RGs in the spinal cord tissue of rat during both spinal cord development (postnatal day 1 to 43 and compared to adulthood) and spinal cord injury. The selected RGs (Table 1) represent common housekeeping genes of basal cell metabolism (*Gapdh*, *Hprt1*, *Mapk6*), and protein translation (*Rpl29*, *Eef1a1*, *Eif2b2*), as well as newly designed RGs (*Gpatch1*, *Gorasp1*, *Cds2*) selected according to the RefGenes tool of the GeneVestigator^®^ database. The stability of the RGs was assessed by the geNorm, NormFinder, and BestKeeper applets. The applets concurred in the rating of the two/three best-rated RGs during development and SCI, respectively. Convenience of the selected RGs was demonstrated by an analysis of the gene expression of two selected target genes with a clear biological context in the spinal cord—*Gfap* and *Glast*—during postnatal development and *Iba1* and *Ed1* after SCI.

## 2. Materials and Methods

### 2.1. Experimental Animals

The experiments were performed with the approval of the National Veterinary and Food Administration of the Slovak Republic, Animal Care Committee of Pavol Jozef Šafárik University in Košice and Review Committee of the University of Milan, in accordance with the European Communities Council (Directive 2010/63/EU) and in compliance with current national legislations. Wistar albino rats were obtained from Velaz (Prague, Czech Republic). Animals were housed under standard laboratory conditions with a 12-h light-dark cycle. Each animal was fed a complete and balanced standard laboratory diet (Peter Miško, Snina, Slovak Republic) and had ad libitum access to food and water. Analyses were performed on male rats at the age of postnatal day 1 (P1), P8, P15, P22, P29, P36, P43, and P120+ (weighing 390 ± 50 g) in the developmental part of the study and on P120+ animals in spinal cord injury experiments (*n* = 3 animals per group/time point).

#### Spinal Cord Injury Models and Tissue Isolation

Two experimental models of spinal cord injury (SCI) were used in the study: mild (minimal spinal cord injury; mSCI) and severe (contusion spinal cord injury; cSCI). In both experimental SCI models, animals were deeply anesthetized with isoflurane (2% maintenance in room air), and partial Th13 laminectomy was performed to expose the dorsal surface of the L3 spinal segment. In cSCI animals (*n* = 6 animals), contusion of the spinal cord was induced by placing a 3.2-mm acrylic rod (weight = 50 g) for 15 min on the dorsal surface of the exposed L3 spinal segment. In mSCI animals (*n* = 6 animals), spinal cord injury was induced by single unilateral incision of the 20 Gauge needle tip to the area of the dorsal root entry zone (950 µm laterally from the medial plane; z = 1150 µm) of the exposed L3 spinal segment. In sham-operated animals (*n* = 6 animals), only laminectomy was performed. The wound was treated with Gentamycine (2 mg/mL) and 0.5% Marcaine (bupivacaine hydrochloride) and sutured in two layers. A systemic dose of Gentamycine (0.1 mg) was administered intramuscularly. During the postsurgical care, animals were inspected regularly, and the bladders were expressed manually twice daily (in groups with cSCI). The severity of SCI was scored daily according to the Basso, Beattie, and Bresnahan (BBB) open field locomotor rating scale, ranging from score 0 (complete paralysis) to score 21 (normal locomotion). Animals were allowed to survive for 24 h or 4 days after surgery (1 or 4 d survival time), anesthetized with an i.p. overdose of Thiopental, and transcardially perfused with cold heparinized saline. The wound was opened, the spinal cord tissue (L3 spinal segment; 20–50 mg) was isolated, briefly washed in saline, placed in TRI Reagent (MRC, Cincinnati, OH, USA), and stored at −80 °C.

### 2.2. Analysis of Gene Expression

#### 2.2.1. RNA Isolation and cDNA Synthesis

Total RNA was extracted by TRI Reagent (MRC, Cincinnati, OH, USA) according to the manufacturer’s instruction. The tissue was homogenized by a sterile disposable plastic pestle directly in the TRI Reagent. The integrity of the isolated RNA was confirmed by agarose gel electrophoresis. The amount of RNA was quantified spectrophotometrically by a spectrophotometer BioSpec-nano (Shimadzu, Kyoto, Japan) and by fluorescent dye RiboGreen using QuantiT™ RiboGreen^®^ RNA reagent kit (Invitrogen, Eugene, OR, USA). In total, 1 μg of RNA was used for reverse transcription by RevertAid reverse transcriptase (Thermo Scientific, Waltham, MA, USA) and anchored oligo dT primer (T_12_VN) according to the manufacturer’s instruction. The resulting RNA/cDNA was stored at −20 °C until quantitative real-time RT PCR (RT-qPCR).

#### 2.2.2. Primer Design and Gene Selection

Nucleotide mRNA sequences of the nine tested housekeeping genes (assigned here as reference genes; RGs) and four genes of interest (target genes, TGs) were obtained from the GenBank^®^ database (http://www.ncbi.nlm.nih.gov). For each gene, several oligonucleotide primers were designed by the PrimerBLAST [22] online primer design tool at NCBI. The specificity check was selected for rat Refseq mRNA and properties were limited to amplified mRNA, i.e., to span two neighboring exons or to bind different exons. Primer pairs (Table 2) with no secondary structures and the lowest delta G were chosen by Unipro UGENE v1.20.0 software [23]. To fulfil the criteria in some genes, the length of the amplicons was allowed to reach 300 bp. The primers for amplification of the TG were designed to bind all known alternatively spliced isoforms.

#### 2.2.3. Quantitative RT-PCR and Normalized Gene Expression

Quantitative real-time RT-PCR (RT-qPCR) with SYBR Green detection of the amplicons was carried out by CFX96 Touch Real Time PCR Detection System (Bio-Rad, Hercules, CA, USA) using Xceed qPCR SG Mix/Lo-ROX (IAB, Prague, Czech Republic) chemistry. Amplification of a single desired product was confirmed by electrophoresis in 2% agarose gel and dissociation analysis (melt curve). The RT-qPCR reaction was performed in a 10-μL volume containing 1× SG Mix, 0.5 μM forward and reverse primer, and 20 ng of template cDNA. The amplification profile was as follows: 95 °C/4 min, 40× (95 °C/8 s, 60 °C/20 s) followed by melt curve analysis. All samples were analyzed in triplicates. The course of amplification, PCR efficiency, and cycle of quantification (Cq) were monitored and evaluated by BioRad CFX Manager 3.1 software. The PCR efficiency and relative gene expression (relative quantity; RQ) was ascertained from standard curve amplification. The samples for standard curve amplification were prepared by serial dilution (4-fold) of the cDNA sample prepared as a mixture of aliquots from all tested samples. Standard curve samples were run in one assay together with the experimental samples and NTC (no template control). The efficiency of the amplification of the primers was within 72% to 101%, with *r*^2^ > 0.993. The technical replicates were averaged and RQ was calculated for each sample and gene. The relative quantity of the TGs was afterwards normalized with the normalization factor (NF). The NF values were calculated by geNorm V3.5 applet as the geometric mean of the most stable RGs.

#### 2.2.4. Reference Gene Stability Testing

The stability of the expression of the tested RGs in the experimental conditions was determined by three highly credible applets, geNorm V3.5 [19], NormFinder v0.953 [21], and BestKeeper v.1 [20]. For an evaluation of the gene expression stability by geNorm and NormFinder, relative RQ values were used. For BestKeeper analysis, non-transformed Cq values and amplification efficiencies were applied. The stability of the RGs during postnatal development and after SCI was evaluated in separate analyses. In the case of SCI, the two different SCI models were also assessed *en bloc* (complex SCI) or individually (mSCI and cSCI only).

The GeNorm applet was used to calculate the Genorm M and Genorm V value. The GeNorm M value represents the gene expression stability value calculated for each RG as the average pairwise variation of the gene with all other tested RGs. The lower the M value is, the better the stability of the gene expression across the analyzed samples. Additionally, the geNorm V value refers to the estimation of the optimal number of RGs sufficient for reliable data normalization. It is based on a pairwise variation of two successive normalization factors (V_n_/V_n+1_). The cut-off value of V_n_/V_n+1_ below 0.15 indicates that no additional (n + 1) reference gene is required.

BestKeeper provides extensive descriptive statistics for variations of each gene across all samples and computes numerous pairwise correlations. We ranked the optimal RGs according to the coefficient of correlation (r) based on the Pearson correlation coefficient and BestKeeper index (geometric mean of Cq values of the candidate RGs). In the case of discrepancies between the results of the tested applets, the standard deviation of the Cq values (Std dev, ±Cq) was also considered.

The NormFinder applet was used to calculate the stability score (S value) for the tested RGs. The merit of the applet is that it takes into account the experimental context and allows for determination of the experimental groups in the sample set. The result is based on intra-group and inter-group variations in the expression of all RGs across all samples. By default, we defined each biological replicate as an individual group. Afterwards, in the additional analyses, we also tested different data management (e.g., merging of controls and sham, merging treatments, or skipping the group labeling).

### 2.3. Data Representation and Statistics

The raw Cq values for each gene are represented in a box-and-whisker plot. The 25th and 75th percentiles are represented as the lower and upper limit of each bar. The upper and lower limit of the whiskers are equal to 1.5 times the interquartile distance. Data were analyzed and graphs were prepared in Excel (Microsoft Office 2016, Microsoft Corporation, Redmond, WA, USA). The differences between experimental groups/time points were analyzed using one-way analysis of variance (ANOVA) followed by the Tukey–Kramer post hoc tests for multiple comparisons. Differences were considered statistically significant if * *p* < 0.05, ** *p* < 0.01, and *** *p* < 0.001.

## 3. Results

The aim of our study was to select and identify stable reference genes (RGs) suitable for the normalization of gene expression data in the rat spinal cord during postnatal development and after spinal cord injury (SCI). Therefore, we tested the stability of the expression of nine selected housekeeping genes in the spinal cord tissue of intact animals at the age of one day (P1) to six postnatal weeks (P43) and in adulthood (P120+) in the developmental part of the study; and in adult animals (P120+) subjected to mild (minimal injury model) and severe (contusion model) SCI. Animals were allowed to survive 1 or 4 days after SCI to cover the most dynamic sub-phases of secondary injury and each experimental group was represented by three individuals (biological replicates). The RGs were evaluated individually for stability during postnatal development and for SCI treatment. In the case of SCI, the stability of RG expression was assessed both complexly for mSCI and cSCI together, as well as independently for each individual model of injury itself.

For the identification of proper reference genes, three mathematical models (geNorm, NormFinder, and BestKeeper) were used. Besides the commonly used housekeeping genes, eukaryotic translation elongation factor 1 alpha 1 (*Eef1a1*), glyceraldehyde-3-phosphate dehydrogenase (*Gapdh*), hypoxanthine phosphoribosyltransferase 1 (*Hprt1*), mitogen-activated protein kinase 6 (*Mapk6*), and ribosomal protein L29 (*Rpl29*), we also tested another four RGs: CDP-diacylglycerol synthase 2 (*Cds2*), eukaryotic translation initiation factor 2B subunit beta (*Eif2b2*), Golgi reassembly stacking protein 1 (*Gorasp1*), and G-patch domain containing 1 (*Gpatch1*). These genes were recommended by the “RefGenes” tool of the GeneVestigator^®^ database, since they showed high expression stability during rat development or after CNS injury among several experiments present in the database. To our knowledge, they have not been used as RG in neuroscience research yet. These newly designed RGs encode for functional proteins and there are no alternative spliced isoforms of these genes in the GenBank^®^ database. According to the “RefGenes” tool, all nine RGs should be expressed at an approximately similar level compared to common genes expressed in the spinal cord, e.g. *Gfap*, *Glast*, or *Iba1*. Two selected target genes (TGs) with a clear biological context in the spinal cord were used in each experiment to validate the suitability of the best scored RGs for normalization of the expression data. We used *Gfap* and *Slc1a3*/*Glast*, as typical genes expressed in the astroglial lineage during spinal cord development in a medium to high level and *Aif1*/Iba1 and *Cd68*/*Ed1* for the monitoring of microglial activation and monocyte infiltration after SCI.

### 3.1. Comparison of the Expressions of the Tested Genes

**Figure 1:**
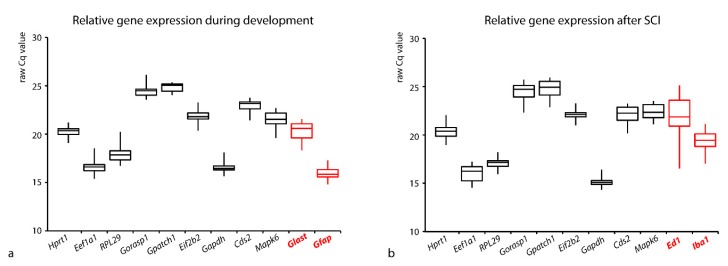
The range of gene expression of the tested reference and target genes in the rat spinal cord during postnatal development (**a**) and after SCI (**b**). The raw Cq values for each gene are represented in the box-and-whisker plot, (**a**) *n* = 24 samples (three biological replicates at P1, P8, P15, P22, P29, P36, P43, and P120+), (**b**) *n* = 21 samples (three biological replicates of intact P120+ controls, sham-operated animals, and animals subjected to mSCI and cSCI for both survival times).

All tested RGs, as well as selected TGs, were expressed across the whole postnatal development and/or after SCI. Amplification of one specific amplification product was confirmed by the dissociation curve of qPCR. Besides the abovementioned stable and unbiased expression of the RGs during experimental conditions, ideal RGs should also be expressed at similar level as the analyzed TGs. In our experiments, the differences between the expression of the tested RGs and TGs were not extensive (Figure 1). The abundance of a gene transcript (template mRNA) in the sample is reflected in RT-qPCR by the Cq value. Although the Cq value does not explicitly necessarily match the absolute quantity of the template (in terms of the intergenic/inter-run comparisons), in a simplified way, the raw Cq value in a given sample reflects the abundance of the individual mRNA.

The mean Cq values of the individual RGs during postnatal development and after SCI ranged from 16.7 to 24.8 and from 15.1 to 24.6, respectively. In both experiments, the lowest mean Cq values (the highest gene expression) were recorded by *Eef1a1* and *Gapdh* and the highest mean Cq values (the lowest gene expression) were observed by *Gorasp1* and *Gpatch1*. However, the differences between the expression (Cq values) of the tested RGs and the analyzed TGs were not so eminent (Figure 1). For example, the mean Cq value of *Glast* during development was a maximum of 4.6 cycles lower compared to the RG with the highest expression and 3.6 cycles higher compared to the RG with the lowest expression (Figure 1a). Similarly, the mean Cq value of *Iba1* in samples in the SCI experiments was approximately 5.2 cycles lower and simultaneously 4.4 cycles higher compared to RGs with the highest and lowest expression, respectively (Figure 1b).

### 3.2. Stability of RGs Expression during Postnatal Development

The stability of the gene expression of the nine tested RGs in the spinal cord during postnatal development was tested on animals at the age P1, P8, P15, P22, P29, P36, P43, and P120+. Small variability of the raw gene expression in the sample set of the development experiment is also obvious from the mean Cq values (Figure 1a). The CV (coefficient of variation) of the calculated RQs (relative quantity) in the RGs ranged from 30.6% (*Gpatch1*) to 66.5% (*Mapk6*). According to geNorm analysis, the majority of the tested RGs showed high stability characterized by rather low M value (M ≤ 0.5 for the five best rated genes) and by the high BestKeeper coefficient of correlation (Table 3). All three used applets favored two genes (*Gapdh* and *Eef1a1*) as the most stable. According to the mean Cq values, these two genes also showed the highest gene expression during the whole postnatal development. A slightly different preference of the most stable RGs would be acquired if the experimental groups were not considered in the NormFinder analysis. In this case, the *Hprt1* gene skips to the second position and the order and stability value of the first four genes would be: *Gapdh* (0.163), *Hprt1* (0.214), *Eef1a1* (0.221), and *Gpatch1* (0.262).

According to the pair variability (V_n_/V_n+1_, Table 3) of the geNorm applet, the calculated V_2/3_ = 0.141 (i.e., V_n_/V_n+1_ ≤ 0.15) shows that the addition of the third RG for the calculation of the normalization factor does not improve the reliability of the normalized data. Therefore, the application of two RGs should be sufficient for reliable normalization of gene expression data during postnatal spinal cord development. Considering the results from the three mathematical applets, the combination of two genes (*Gapdh* and *Eef1a1*) should fulfill the criteria for proper normalization of gene expression data. Moreover, since the *Gapdh* gene is involved in cell metabolism and *Eef1a1* in protein translation, it is not expected that the highly correlated results of the three applets are due to the co-regulated gene expression.

**Figure 2:**
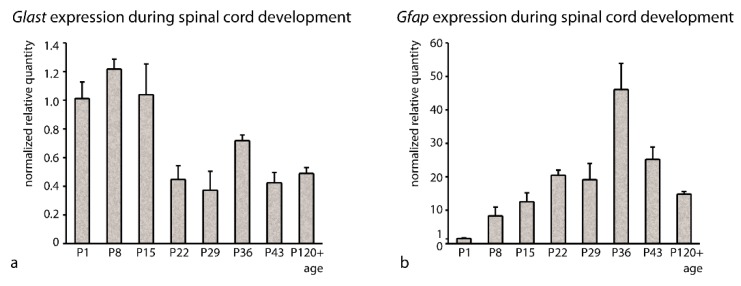
Relative gene expression of *Glast* (**a**) and *Gfap* (**b**) in the spinal cord during rat postnatal development. Normalized relative gene expression at P1, P8, P15, P22, P29, P36, P43, and P120+ (adulthood) represents the average of three biological replicates ± standard deviation (SD). The expression of the genes is normalized by the normalization factor based on the expression of *Gapdh* and *Eef1a1*.

The suitability of the best rated reference genes (*Gapdh*, *Eef1a1*) was also confirmed by analysis of the *Gfap* and *Glast* gene expression in the spinal cord (Figure 2) during the same intervals of rat postnatal development. Expression of the TGs was normalized by the normalization factor computed from the expression of *Gapdh* and *Eef1a1*. As expected, expression of *Gfap* increased during maturation of the spinal cord occurring in the first postnatal weeks. Its expression gradually increased until P22 and afterwards remained more or less stable. This expression pattern reflected extensive differentiation and maturation of astrocytes during early postnatal development. Significantly increased expression of *Gfap* at P36 (*p* < 0.001 according to ANOVA, Tukey–Kramer multiple comparisons test) could be explained by unintended contamination of the sample by dorsal root ganglion and/or periphery nerve, since we detected a significantly increased amount of *Gfapβ* isoform in the P36 samples, which is commonly present in PNS [25]. On the other hand, a very different expression pattern was shown with *Glast*, which is associated with the phenotype of radial glial progenitors and young undifferentiated astrocytes during early development and is present in mature astrocytes only in restricted areas of the spinal cord during late postnatal development and in adulthood.

### 3.3. Stability of RGs Expression after SCI

Two different experimental models of injury were used for identification of the least variable genes in the adult animals, minimal SCI (mSCI) and severe contusion injury (cSCI). The expression of the RGs and TGs was monitored one and four days after each surgery (mSCI + 1 d, mSCI + 4 d, cSCI + 1 d, cSCI + 4 d). Neither of the experimental models caused lethality of the animals during the tested interval, but the cSCI resulted in paraplegia of the animals (maximum BBB scores of 0–2). In the case of mSCI, no obvious disability affecting the movement of the hind limbs was observed (BBB score = 21). One can assume that even minimal SCI should result in tissue damage accompanied with several responses at various levels in the injured spinal cord (inflammation, astrogliosis, activation of microglia, etc.) and these processes may also affect the expression of both tested RGs, as well as TGs. Depending on the severity of injury, the effect may be different; therefore, the stability of the RGs’ expression was considered either for both injuries together or individually for mSCI and for cSCI.

#### 3.3.1. Stability of RGs’ Expression after SCI Examined *en bloc* (cSCI and mSCI Altogether)

Compared to the developmental experiment, slightly higher variability of the gene expression in the spinal cord was observed after the SCI, as is readily apparent from the mean Cq values (Figure 1b). The highest CV of the raw RQs is obvious mainly in *Gorasp1*, *Gpatch1*, and *Cds2* (70.2%, 67.7%, and 66%) while the lowest was in *Gapdh* (27.6%).

According to the geNorm M value, there are still four RGs possessing an M value ≤ 0.5 (Table 4). As expected, the ranking of the most stably expressed RGs was also changed (Table 4) in comparison to the developmental experiment. Higher gene expression variability also gave rise to an increased number of RGs required for reliable data normalization. According to the geNorm pair variability (V_2/3_ = 0.164 > 0.15 > V_3/4_ = 0.118), at least three genes should be used for accurate normalization of gene expression. Remarkably, the individual mathematical models suggested an identical combination of the three uppermost stable RGs, *Eif2b2*, *Gapdh*, and *Gorasp1*. On the other side, in this case, a significantly inferior rating of *Eef1a1* and *Rpl29* after SCI was recorded in comparison to their high ranking during development.

Regarding the NormFinder analysis, there was no noticeable effect of the experimental group management on the evaluation. The ranking of the genes at the top positions was almost the same if different experimental groups were labeled in the applet. The only noticeable rearrangement of ranking occurred after merging all controls and SCI samples into two groups but only at the third and later positions.

#### 3.3.2. Stability of RGs’ Expression after mSCI and cSCI Examined Separately

The impact of the individual SCI (mSCI or cSCI) on the stability of RGs expression was also assessed separately. The effects of both types of injury at the tissue level differed diametrically. After minimal injury, only a minor part of the neural tissue was damaged, which did not result in extensive inflammation, while the cSCI irreversibly destroyed the architecture of the lesioned segment and the negative effects of the massive inflammation spread thorough the neural tissue. According to our results, an independent evaluation of both models of SCI resulted in two different sets of candidate RGs with stable expression after the individual injury (Table 5 and Table 6). Moreover, the validation applets did not reach an entire agreement.

In the case when specimens with mSCI were evaluated separately (Table 5), the three best rated genes were as follows: (i) *Eif2b2*, *Gapdh*, and *Eef1a1* (suggested by geNorm and NormFinder); or (ii) *Eif2b2*, *Gapdh*, and *Gorasp1* (suggested by BestKeeper). The declared stability of *Gapdh* and *Eef1a1* was in agreement with the low variance of the best rated genes during spinal cord development. Nevertheless, according to the common agreement of the three applets, the gene with potentially the highest stability of expression after mSCI seemed to be *Eef2b2*, which can be safely combined with *Eef1a1* or/and *Gapdh* for reliable normalization of gene expression data in the spinal cord after mSCI.

The assessment of the individual effect of cSCI on the stability of RGs’ expression (Table 6) revealed a mixed bag of results depending on the applet used. Thus, the three best rated genes were: (i) *Hprt1*, *Gorasp1*, and *EiF2b2* (geNorm); (ii) *Gapdh*, *EiF2b2*, and *Hprt1* (NormFinder); or (iii) *Gapdh*, *Mapk6*, and *Gpatch1* (BestKeeper). However, the high values of the coefficient of correlation in the BestKeeper results were combined with the worst values of the Cq variability (standard deviation, Table 6), which indicated instable expression of the genes. Similarly, *Mapk6, Gorasp1*, *Gpatch1*, and *Cds2* possessed high overall RQ variability in the SCI sample set; therefore, the next candidates for RGs with stable gene expression were *EiF2b2* and *Hprt1*. In this regard, the supposed RGs for cSCI normalization are *EiF2b2*, *Gapdh*, and *Hprt1* or *Gorasp1*.

It can be assumed that the splitting of the complex SCI treatment into separate models/analyses resulted in altered variation of individual RGs’ expression and therefore altered the set of stably expressed RGs. The altered variation is reflected also in the geNorm pair variabilities in both SCI models; the V_2/3_ values below 0.15 (mSCI V_2/3_ = 0.087 and cSCI V_2/3_ = 0.131, respectively) suggest that the application of only two RGs should be sufficient for reliable normalization of the expression data.

**Figure 3:**
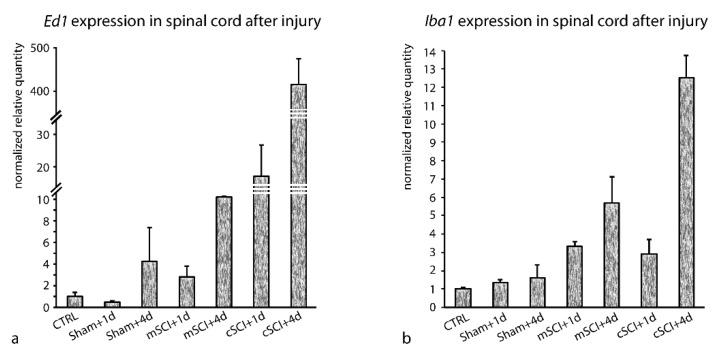
Relative gene expression of *Ed1* (**a**) and *Iba1* (**b**) in the rat spinal cord after spinal cord injury (mSCI or cSCI). The injury was performed on adult animals and the survival times were +1 and +4 days. Normalized relative gene expression represents an average of three biological replicates ± SD. Expression of the TGs was normalized by a normalization factor based on the expression of *Eif2b2*, *Gapdh*, and *Gorasp1*.

The suitability of the best rated reference genes from the *en bloc* analysis (*Eif2b2*, *Gapdh*, and *Gorasp1*) for data normalization was confirmed also by the analysis of the *Ed1* and *Iba1* gene expression in the spinal cord (Figure 3) subjected to either the minimal or contusion injury model with 1- or 4-day survival time. Our results showed overexpression of *Ed1* and *Iba1* predominantly after cSCI.

In both models, the effect of injury was more prominent on the fourth day after SCI. There was also a significant difference in the amplitude of the response between the TGs. The upregulation of *Iba1* (3- to 6-fold) compared to the intact spinal cord reflected the activation and proliferation of microgial cells in the direct response to the injury. Furthermore, striking overexpression of *Ed1* (up 415-fold) compared to the intact spinal cord indicated, besides the activation of microglia, the infiltration of ED1-expressing (CD68-positive) monocytes/macrophages from disrupted blood vessels.

## 4. Discussions

Reference genes are indispensable components of each gene expression analysis by means of real-time RT-PCR. Normalization of the qPCR data by internal controls (RGs) is the most highly accepted and most frequently used method for compensation of non-biological variations across the compared sample set. Alternative approaches for normalization rely on accurate measurement of the sample size in the compared samples (tissue weight, volume of cell number), accurate quantification of the total RNA input, or quantification of external controls (spiking the samples with exogenous nucleic acid) (reviewed in [2]). However, these procedures are inconvenient for routine analysis (especially with many samples) or infeasible in certain experimental designs, and the reliability faces an even higher rate of criticism.

Unfortunately, as was proven many times in the past, ideal and universal control gene(s), which is/are stably expressed across all cell types or under different conditions, do not exist [5,9,26]. Therefore, it is recommended that the combination of several validated RGs is employed to minimize the inherent variation in their expression [19]. In the quest for optimal RGs, stably expressed in the particular type of cells/tissue in given conditions, several algorithms were developed. The highest popularity acquired the “ready-to-use” user-friendly applets geNorm, NormFinder, and BestKeeper [19,20,21]. In spite of their drawbacks and occasional inconsistency in results, when compared side by side [15,27], these applets represent straightforward and valuable tools for routine RG testing. The source of the abovementioned inconsistency between the results of the applets lies in their unique algorithms assuming relative stability of the tested gene expressions. Anyhow, since these methods select the best RGs from a set of selected candidates, one can assume that the better the candidates are, the better the result should be that is acquired.

Although real-time PCR technology was introduced 25 years ago [28], validation of RGs’ stability in experimental conditions is still not commonplace in most experimental studies. On the other hand, there are several methodology-oriented papers dealing with the selection of optimal reference gene(s) in the particular cell type/tissue under certain conditions. Experimental studies that have determined the selection of stably expressed RGs in neural tissue are summarized in Table 7. To the best of our knowledge, a relevant study focused on the selection of appropriate RGs in the rat spinal cord during development or after spinal cord injury is still missing.

### 4.1. Selection of Candidate RG Set

The aim of our study was to select and validate proper RGs applicable for normalization of qPCR data in the spinal cord (i) during postnatal development (since the early postnatal development–P1 to the adulthood) and (ii) after injury of the adult spinal cord. It is obvious that the quality of normalized expression levels is entirely dependent on the quality of the normalizer, i.e., the appropriate reference gene(s). In our study, we tested the stability of the expression of nine candidate RGs, including five common RGs and four newly designed RGs. The selected common RGs—*Eef1a1*, *Gapdh*, *Hprt1*, *Mapk6*, and *Rpl29*—showed stable expression in nervous tissue in previous studies; *Rpl29* and *Hprt1* in a spared nerve injury model of neuropathic pain in the rat in the dorsal horn of the spinal cord and dorsal root ganglion (DRG), respectively [16]; and *Mapk6*, *Gapdh*, and *Hprt1* in DRG after peripheral nerve injury [24] or in the dorsal horn after inflammatory injury [18]. We also included in the analysis four new RGs, which have not been used in nervous tissue of rat before: *Cds2* (CDP-diacylglycerol synthase 2), *Eif2b2* (eukaryotic translation initiation factor 2B subunit beta), *Gorasp1* (Golgi reassembly stacking protein 1), and *Gpatch1* (G-patch domain containing 1). The genes *Cds2*, *Gorasp1*, and *Gpatch1* were newly designed upon the recommendation of the “RefGenes” tool of the GeneVestigator^®^ database [5]. Both genes, *Eef1a1* and *Eif2b2*, were selected as the most promising members of the respective translation factor family by the “Development” tool of the GeneVestigator platform [29]. The newly designed candidate RGs (*Cds2*, *Gorasp1*, *Gpatch1*, and *Eif2b2*) represent true housekeeping genes orthologous to human housekeeping genes affirmed by RNA-seq analysis of several human tissues [3]. According to the study, only *Gorasp1* fulfilled the criteria proposed for a proper calibration gene.

In addition, with regard to the expression level, all selected RGs should be suitable for RT-qPCR analysis in the spinal cord, since their expression levels (Cq values) are similar to the tested target genes, representing the common genes transcribed in the spinal cord.

### 4.2. Stability of Expressed Genes during Rat Postnatal Development

Although the most prominent developmental processes, such as the formation of the neural tube and neurogenesis, occur during embryonic period, the spinal cord of rodents undergoes important developmental changes even during postnatal life. During the postnatal period, the spinal cord triples its length [30] and glial progenitors intensively proliferate both inside the central canal [31] and in the surrounding gray and white matter [32]. Differentiating neurons change their neurotransmitter profile and newly formed oligodendrocytes extensively myelinate nerve fibers originating from both spinal neurons as well as those of descending nerve tracts (for a review, see [33]). Despite the huge bulk of processes, our results show, overall, small variability of candidate RGs’ expression during the whole tested postnatal development. The obtained geNorm M values, which were, for several RGs, ≤0.5, show relatively high stability of the tested genes and indicate a homogenous sample set [34], as expected in untreated samples comprising only one kind of tissue, i.e., spinal cord. All three employed applets favored *Gapdh* and *Eef1a1* as the most suitable RGs in the tested sample set. Moreover, since both genes belong to different GO (gene ontology) categories, co-regulated gene expression is not expected. According to the sample variability (V_2/3_ = 0.141 < 0.15), the employment of two genes should be sufficient for data normalization. On the other side, the 0.15 cut-off value is the only rule of thumb and the application of at least three RGs is appreciated [19].

As mentioned previously, there is no relevant study dealing with the stability of RGs in the rat spinal cord during (postnatal) development. To our knowledge, the only relevant study dealing with the stability of RG expression during development [15]) suggested *Mrpl10* and *Ppia* as the best RGs in the mouse spinal cord tissue while *Gapdh* was omitted from the analysis due to high CV values (high variability of SQ values in the experiment). In similar studies on brain tissue, a stable expression pattern of *Gapdh* was detected in the rat somatosensory cortex [35] and in the murine neocortex [36] during postnatal development, and in the rat cortex and hippocampus during aging [37]. On the other hand, *Gapdh* showed a rather high variability in most mouse brain regions in the first postnatal week and also in adulthood [38]. Obviously, there are numerous reasons that may lead to these apparently contradictory results, e.g., different animal species, different areas within the CNS, as well as different sample sets (different age/age range) or different sets of genes assessed in the compared studies. However, biologically related tissues should share a significantly higher degree of overlapping of the stably expressed genes than the global mean. Moreover, similar expression profiles (i.e., stable expression) of orthologous sequences could be extrapolated from validated species to an unknown one [5]. Although normalization of gene expression data by *Gapdh* is often subjected to criticism in the literature, mainly due to its variable expression, e.g., [4,6,15,39], it showed extraordinary stability in our study. On the other hand, an unresolvable drawback of the *Gapdh* gene is the tremendous amount of pseudogenes: 67 in human and 197 in the mouse genome [40], which are mainly intronless and a similar size to the “original” gene. However, there are several recent studies admitting *Gapdh* as a proper RG for data normalization in neural tissue [24,35,36]. Therefore, patient validation of its stability in the experiment and careful sample preparation disposing of any gDNA contamination is essential if *Gapdh* was used for data normalization.

In our hands, the stable expression of *Gapdh* and *Eef1a1* was shown to be valuable for the normalization of gene expression in the spinal cord during postnatal development. *Gapdh/Eef1a1*-normalized expression of *Gfap* (increase of *Gfap* mRNA until P22) and *Glast* (decline of *Glast* mRNA on P22) in the spinal cord during the first postnatal weeks reflected extensive differentiation and maturation of astrocytes during the early postnatal development. As shown in our previous study, spinal cord gray matter is fully settled with maturing GFAP+ astrocytes after P15 [41]. On the other hand, *Glast* is associated with the phenotype of radial glial progenitors and young undifferentiated astrocytes during early development and is present in mature astrocytes only in restricted areas of the spinal cord during late postnatal development and in adulthood [41].

### 4.3. Stability of RGs Expression after SCI

Traumatic events resulting in damage of the spinal cord represent a severe health condition with an unfavorable long-term prognosis, despite all the progress in neurobiological research. In order to study the regenerative processes and various therapeutic approaches, a wide range of experimental SCI models on rodents with various impacts on the architecture of the nervous tissue were developed. Similarly, as in other fields of neurosciences, in neuroregenerative research, proper RGs should be identified for RT-qPCR analyses. Therefore, in our study, we used two models of injury, minimal SCI (mSCI) mild “provocative” injury aimed to study endogenous neuroregenerative processes of the spinal cord tissue, and severe contusive injury (cSCI), destroying the architecture of the spinal cord. These markedly different models of injuries were chosen to cover the scale of injuries routinely used for study of the regenerative potential of the adult spinal cord, and measurement of the expression one and four days after SCI to cover the most dynamic sub-phases of secondary injury.

If the effect of both models of SCI on RG expression was considered complexly, the genes *Eif2b2*, *Gapdh*, and *Gorasp1* showed the most stable expression. This assumption was supported by the consistent ranking of the genes by all three used applets. An altered ranking of the RGs was acquired when the two different models of injury were considered individually. In the mSCI model, the three best rated genes were *Eif2b2* and *Eef1a1*, as well as *Gapdh* or *Gorasp1*, depending on the applet used. The overlapping of the best rated genes (*Eef1a1* and *Gapdh*) in the mSCI model and in the developmental study is in accordance with the expected low impact of the mild injury on the “normal” gene expression. Although, in the cSCI model, some discrepancy in the RGs’ ranking was obtained, we assume that the least variable genes are *Eif2b2*, *Gapdh*, and *Hprt1* or *Gorasp1*. In all cases, either in individual mSCI/cSCI or in complex SCI evaluation, at least four genes possessed a calculated M value lower than 0.5, which points to the relatively high expression stability [34].

Taken together, *Eif2b2* displayed a very high stability of expression in the spinal cord after both models of SCI. Depending on the extent of the injury, we recommended to append to the calculation of the normalization factor the additional expression of the genes *Gapdh* and *Gorasp1* or *Hprt1* in experiments employing the more extensive injury, or *Gapdh* and *Eef1a1* in mild injury models.

So far, a relevant comprehensive study of RGs stability in the spinal cord of the rat (or other mammals) after spinal cord injury was not published. However, there are several reports (Table 7) dealing with an evaluation of the stability of RG expression in the spinal cord (e.g., in the dorsal horn) or in the dorsal root ganglia after different models of nerve injury and neuropathic pain, as well as in peripheral nerve injury [24,42], spared nerve injury [16], and nerve root compression [39]. There are also few studies validating proper RGs expressed in the brain after traumatic brain injury [43,44,45,46]. The genes *Gpadh* and *Hprt1*, which were found to be stably expressed in our study, either share the high ranking, e.g., [16], or are found to have a variable expression pattern, e.g., [39]. The reasons for this inconsistency could be similar to those in the development study, i.e., the employment of different species, different regions of the nervous system, diverse sample set, or more or less fitted set of genes used in those studies.

Naturally, one of the most prominent processes occurring after the spinal cord injury is inflammation. The immune reaction inside the nervous tissue is mediated by microglia and a variety of other immune cells infiltrating the site of the injury (reviewed in [47]). In our study, we showed that the onset of inflammation in the spinal cord after injury may be easily monitored and quantified by RT-qPCR (expression of inflammatory markers, *Ed1* and *Iba1*) in the case when the RGs are properly selected. We showed that the expression of *Ed1* and *Iba1* is significantly increased after SCI. Moreover, a massive increase of the *Ed1* expression in sham controls or mSCI may indicate an improper technique was used during surgery. This gives an interesting clue for the easy testing of properly performed minimal SCI.

## 5. Conclusions

An accurate and reliable gene expression study by means of real-time RT-PCR necessitates knowledge of stably expressed reference genes (RGs). Since generally applicable RGs do not exist, it is inevitable that RGs; stability of expression is assessed in any experimental context. In our study, we selected and validated stably expressed RGs in the rat spinal cord, namely during postnatal development (from the first postnatal day to adulthood) or after spinal cord injury. In the set of nine selected candidate RGs, we found *Gapdh* and *Eef1a1* to be the most valuable for the normalization of gene expression data during postnatal development, and *Eif2b2* in combination with *Eef1a1* and/or *Gapdh* in minimal SCI or *Gapdh* and *Gorasp1* or *Hprt1* in more severe experimental models.

## Figures and Tables

**Table 1:** Candidate reference genes analyzed in the study.

Gene Symbol	Gene Name	Main Function
*Cds2*	CDP-diacylglycerol synthase 2	Glycerophospholipid metabolism and phosphatidylinositol signaling system
*Eef1a1*	eukaryotic translation elongation factor 1 alpha 1	Protein synthesis
*Eif2b2*	eukaryotic translation initiation factor 2B subunit beta	Protein synthesis
*Gapdh*	glyceraldehyde-3-phosphate dehydrogenase	Glycolysis
*Gorasp1*	golgi reassembly stacking protein 1	Golgi apparatus structure.
*Gpatch1*	G patch domain containing 1	Nucleic acid-binding protein
*Hprt1*	hypoxanthine phosphoribosyltransferase 1	Metabolic salvage of purines
*Mapk6*	mitogen-activated protein kinase 6	Protein kinase
*Rpl29*	ribosomal protein L29	Component of ribosomes (60S subunit)

**Table 2:** Nucleotide sequences of the primers used in RT-qPCR.

Gene Symbol	Acc. No	Sequence for	Sequence Rev	Length (bp)
Reference Genes				
*Cds2*	NM_053643	TGGCTGGAAAACCATGAGGAT	GGTACTGGCAGTCAAAGCGA	185
*Eef1a1*	NM_175838	TGCTGGAGCCAAGTGCTAAT	GTGCCAATGCCGCCAATTTT	181
*Eif2b2*	NM_032058	ATCCGCAGAGAGGGTAGGAG	GTGCCTTCCAGTTCCACTAGC	260
*Gapdh* ^1^	NM_017008	AGACAGCCGCATCTTCTTGT	TGATGGCAACAATGTCCACT	142
*Gorasp1*	NM_019385	CTGAAGGCTAATGTGGAGAAG	CCAACAATGTAGTCTGTGTAAGG	239
*Gpatch1*	NM_001106246	GGACCAGCCATCTTCTTGGA	TCTCTCTCGGGTTCTTTGTGA	226
*Hprt1*	NM_012583	TGCTGGTGAAAAGGACCTCTC	AGATTCAACTTGCCGCTGTCT	192
*Mapk6* ^2^	NM_031622	TAAAGCCATTGACATGTGGG	TCGTGCACAACAGGGATAGA	129
*Rpl29*	NM_017150	AGTCCAAGAACCACACCACA	ATTCGTATCTTTGTGACCGGGG	84
Target Genes				
*Aif1 (Iba1)*	NM_017196	CCTCATCGTCATCTCCCCAC	CTCCATGTACTTCGTCTTGAAGG	214
*Cd68 (Ed1)*	NM_001031638	TGGTTCCCAGCCATGTGTTC	TCTGATGTCGGTCCTGTTTG	209
*Gfap*	NM_017009	CACTCAGTACGAGGCAGTGG	ACTCAAGGTCGCAGGTCAAG	176
*Slc1a3 (Glast)*	NM_019225	GACCTCCTCAAGTTCTGCCA	ATCTGGTGATGCGTTTGTCC	83

^1^ adopted from [24], ^2^ adopted from [18].

**Table 3:** Stability of gene expression and ranking of the tested reference genes in the spinal cord during rat postnatal development. The ranking of nine RGs according to stability values and coefficient of correlation calculated by the geNorm, NormFinder, and BestKeeper applets. In NormFinder, the three biological replicates at each timepoint (P1, P8, P15, P22, P29, P36, P43, and P120+) represent an individual experimental group.

	geNorm			NormFinder		BestKeeper		
Gene Rank	Gene	geNorm M	geNorm V	Gene	S Value	Gene	Coeff of Corr. [r]	Std Dev [±Cq]
1	*Eef1a1/Gapdh*	0.378	---	*Gapdh*	0.196	*Eef1a1*	0.935	0.57
2				*Eef1a1*	0.222	*Gapdh*	0.891	0.49
3	*Rpl29*	0.431	0.141	*Gpatch1*	0.231	*Rpl29*	0.889	0.58
4	*Hprt1*	0.474	0.113	*Hprt1*	0.270	*Hprt1*	0.789	0.36
5	*Gpatch1*	0.502	0.095	*Rpl29*	0.290	*Gorasp1*	0.767	0.56
6	*Gorasp1*	0.537	0.088	*Gorasp1*	0.312	*Mapk6*	0.703	0.65
7	*Eif2b2*	0.573	0.082	*Cds2*	0.355	*Gpatch1*	0.669	0.33
8	*Cds2*	0.610	0.079	*Mapk6*	0.400	*Eif2b2*	0.646	0.58
9	*Mapk6*	0.643	0.073	*Eif2b2*	0.407	*Cds2*	0.590	0.49

**Table 4:** Stability of gene expression and ranking of the tested reference genes in the rat spinal cord after spinal cord injury (cSCI + mSCI). The ranking of nine RGs according to stability values and coefficient of correlation calculated by geNorm, NormFinder, and BestKeeper applets. In NormFinder, each biological replicate (intact control, sham control, mSCI, and cSCI with 1 and 4 days of survival time) represents an individual experimental group.

	geNorm			NormFinder		BestKeeper		
Gene Rank	Gene	geNorm M	geNorm V	Gene	S Value	Gene	Coeff of Corr. [r]	Std Dev [±Cq]
1	*Eif2b2/Gapdh*	0.340	---	*Eif2b2*	0.199	*Gapdh*	0.881	0.36
2				*Gapdh*	0.201	*Eif2b2*	0.879	0.38
3	*Gorasp1*	0.453	0.164	*Gorasp1*	0.313	*Gorasp1*	0.825	0.67
4	*Hprt1*	0.498	0.118	*Gpatch1*	0.313	*Gpatch1*	0.813	0.70
5	*Gpatch1*	0.538	0.104	*Hprt1*	0.319	*Cds2*	0.720	0.63
6	*Mapk6*	0.555	0.080	*Mapk6*	0.352	*Mapk6*	0.717	0.67
7	*Cds2*	0.565	0.069	*Cds2*	0.379	*eEF1a1*	0.690	0.78
8	*Eef1a1*	0.625	0.091	*Eef1a1*	0.421	*Hprt1*	0.658	0.53
9	*Rpl29*	0.689	0.094	*Rpl29*	0.457	*Rpl29*	0.212	0.55

**Table 5:** Stability of gene expression and ranking of the tested reference genes in the rat spinal cord after minimal spinal cord injury (mSCI). The ranking of nine RGs according to stability values and coefficient of correlation calculated by the geNorm, NormFinder, and BestKeeper applets. In NormFinder, each biological replicate (intact control, sham control, mSCI with 1 and 4 days of survival time) represents an individual experimental group.

	geNorm			NormFinder		BestKeeper		
Gene Rank	Gene	geNorm M	geNorm V	Gene	S Value	Gene	Coeff of Corr. [r]	Std Dev [±Cq]
1	*Eif2b2/Gapdh*	0.261	---	*Eif2b2*	0.162	*Gorasp1*	0.951	0.81
2				*Eef1a1*	0.187	*Eif2b2*	0.945	0.46
3	*Eef1a1*	0.281	0.087	*Gapdh*	0.211	*eEF1a1*	0.916	0.52
4	*Hprt1*	0.361	0.104	*Mapk6*	0.266	*Gpatch1*	0.915	0.80
5	*Mapk6*	0.410	0.085	*Hprt1*	0.277	*Gapdh*	0.888	0.37
6	*Gorasp1*	0.454	0.080	*Gorasp1*	0.299	*Mapk6*	0.845	0.59
7	*Gpatch1*	0.485	0.070	*Gpatch1*	0.307	*Cds2*	0.844	0.67
8	*Cds2*	0.508	0.061	*Cds2*	0.328	*Hprt1*	0.821	0.49
9	*Rpl29*	0.576	0.084	*Rpl29*	0.428	*Rpl29*	0.151	0.40

**Table 6:** Stability of the gene expression and ranking of the tested reference genes in the rat spinal cord after contusion spinal cord injury (cSCI). The ranking of nine RGs according to stability values and coefficient of correlation calculated by the geNorm, NormFinder, and BestKeeper applets. In NormFinder, each biological replicate (intact control, sham control, cSCI with 1 and 4 days of survival time) represents an individual experimental group.

	geNorm			NormFinder		BestKeeper		
Gene Rank	Gene	geNorm M	geNorm V	Gene	S Value	Gene	Coeff of Corr. [r]	Std Dev [±Cq]
1	*Hprt1/Gorasp1*	0.358	---	*Gapdh*	0.202	*Gapdh*	0.845	0.27
2				*Eif2b2*	0.260	*Mapk6*	0.838	0.78
3	*Eif2b2*	0.405	0.131	*Hprt1*	0.268	*Gpatch1*	0.817	0.81
4	*Gpatch1*	0.491	0.133	*Gpatch1*	0.334	*Cds2*	0.787	0.81
5	*Mapk6*	0.531	0.104	*Mapk6*	0.358	*Eif2b2*	0.779	0.35
6	*Gapdh*	0.555	0.086	*Gorasp1*	0.373	*Gorasp1*	0.773	0.71
7	*Cds2*	0.585	0.083	*Eef1a1*	0.475	*Hprt1*	0.768	0.53
8	*Eef1a1*	0.660	0.102	*Cds2*	0.484	*eEF1a1*	0.496	0.67
9	*Rpl29*	0.744	0.110	*Rpl29*	0.494	*Rpl29*	−0.112	0.60

**Table 7:** Reference genes validation studies performed in the nervous tissue of the mouse, rat, and human. PNI—peripheral nerve injury, SNI—spared nerve injury, TBI—traumatic brain injury, mESC—mouse embryonic stem cells.

Experimental Condition	Tissue	Species	Tested Genes	Best Rated RGs	Reference
development	spinal cord, brain (cerebellum)	mouse, C57BL/6J	*Actb*, *Gapdh*, *Hsp60*, *Mrpl10*, *Pgk1*, *Ppia*, *Rpl13a*, *Rps26*, *Sdha*, *Tbp*	*Mrpl10*, *Ppia*	[15]
development	brain (somatosensory cortex, visual cortex)	rat, Wistar	*Gapdh*, *Hprt1*, *Kif5c*, *Ospb*, *Rn18s*, *Rps18*, *Tfr1*, *Uqcrfs1*, *Ywhaz*	*Ywhaz*, *Uqcrfs1* *Gapdh*, *Tfr1* *Osbp*	[35]
development, in vitro differentiation	brain (neocortex), cell culture—mESC	mouse, C57BL/6	*18S rRNA*, *Actb*, *Gapdh*, *Hprt1*, *RpII*	*Gapdh* *Hprt1*	[36]
development	brain (different parts)	mouse, CD-1	*18s rRNA*, *B2m*, *Gapdh*, *Gusb*, *Pgk1*, *Tfrc*	*Pgk1*	[38]
aging, dietary restriction, glucocorticoid treatment	brain (cortex, hippocampus)	rat, Wistar	*18S rRNA*, *Actb*, *Gapdh*, *Cypb*	*Actb*, *Gapdh*	[37]
PNI	dorsal root ganglia	rat, Sprague-Dawley	*18s rRNA*, *Act*, *Gapdh*, *Hprt1*, *Mapk6*, *Tubb3*, *Tubb5*	*Mapk6*, *Gapdh*	[24]
PNI	sciatic nerve, dorsal root ganglia	rat, Sprague-Dawley	*18S rRNA*, *Actb*, *Ankrd27*, *CypA*, *Gapdh*, *Hprt1*, *Mrpl10*, *Pgk1*, *Rictor*, *Tbp*, *Ubc*, *Ubxn11*, *Ywhaz*	*Mrpl10*, *Tbp*	[42]
SNI	spinal cord, dorsal root ganglia	rat, Sprague-Dawley	*18S rRNA*, *Actb*, *Gapdh*, *Hmbs*, *Hprt1*, *Rpl13a*, *Rpl29*	*Rpl29*, *Rpl13a* *Hprt1*, *Actb*	[16]
neuropathic pain	dorsal root ganglia	rat, Sprague-Dawley	*Actb*, *Gapdh*, *Hmbs*, *Rpl3*, *Rpl19*, *Rpl29*	*Rpl29*, *Rpl3*	[39]
inflammatory injury	spinal cord	rat, Sprague-Dawley	*Actb*, *B2m*, *Hprt1*, *Mapk6*	*Actb*, *B2m*, *Hprt1*, *Mapk6*	[18]
TBI	brain	mouse, CD-1	*18S rRNA*, *Actb*, *B2m*, *Gapd*, *S100b*	*18S rRNA*, *Gapdh*	[43]
TBI	brain (cortex, hippocampus)	rat, Sprague-Dawley	*B2m*, *Gapdh*, *Gusb*, *Hprt1*, *Tbp*, *Sdha*	*Hprt1*, *Sdha*, *Gusb* *B2m*, *Tbp*, *Gapdh*	[44]
TBI	brain (hippocampus, parietotemporal cortex)	rat, Sprague-Dawley	*18S rRNA*, *Actb*, *Cyca*, *Gapdh*	*Actb*, *Ppia*	[45]
TBI, aging	brain (hemispheres)	mouse, C57BL/6N	*18S rRNA*, *Actb*, *B2m*, *Gapdh*, *Hprt1*, *Pbgd*, *Ppia*, *S100b*	*Hprt1*, *Ppia*	[46]
cancer (astrocytoma)	cell culture—astrocytoma	human	*B2M*, *CYC1*, *GAPDH*, *HMBS*, *HPRT1*, *RPL13a*, *SDHA*, *TBA*, *YWHAZ*	*GAPDH*, *RPL13A*, *CYC1*	[48]
cancer (gliomas)	cell culture—glioma	human	*ACTB*, *GAPDH*, *POLR2A*, *RPL13A*, *SDHA*, *TBP*	*ACTB*, *SDHA*	[49]
in vitro (differentiation0	cell culture—Oligodendrocytes	rat, Wistar	*18S rRNA*, *Actb*, *Cyca*, *Gapdh*, *Hmbs*, *Hprt1*, *Pgk1*, *Rpl13A*, *Tbp*, *Ywhaz*	*Cyca*, *Pgk1*, *Rpl13A*, *Ywhaz*	[50]
in vitro (Borna disease virus infection)	cell culture—primary cortical neurons	rat, Sprague-Dawley	*18S rRNA*, *Actb*, *Arbp*, *B2m*, *Gapdh*, *Hprt1*, *Ppia*, *Rpl13a*, *Tpp*, *Ywhaz*	*Arbp*, *Actb*	[51]
in vitro (treatment with carbon monoxide)	cell culture—cortical astrocytes	mouse, C57BL/6	*Actg1*, *Gapdh*, *Hprt1*, *Pgk1*, *Ppia*, *Rn18s*, *Sdha*, *Tbp*	*Gapdh*, *Ppia*	[52]
Disease—neurodegenerative diseases	brain (prefrontal cortex, cerebellum)	human	*ACTB*, *ATP5B*, *B2M*, *CYC1*, *EIF4A2*, *GAPDH*, *HMBS*, *HPRT1*, *PPIA*, *PUM1*, *RPL13*, *SDHA*, *TBP*, *TOP1*, *UBE2D2*, *UBC*	*UBE2D2*, *CYC1*, *RPL13*	[7]
Disease—epilepsy	brain (neocortex temporal lobe)	human	*ACTB*, *B2M*, *CYPA*, *GAPDH*, *HPRT1*, *MAP*-*2*, *MRPL*, *NNE*, *SDHA*, *SYP*, *TBP*, *UBC*	*SYP*, *NSE*, *MRPLl*	[53]
Disease—neurodegenerative disorders	CNS (brain, spinal cord)	human	*AARS*, *ATP5E*, *BECN1*, *CSNK2B*, *DCTN2*, *GAPDH*, *GAPVD1*, *OSBP*, *QARS*, *NAT5*, *TUBB*, *XPNPEP1*	*XPNPEP1*	[54]
Neuroplasticity—morphine addiction	brain (caudate putamen, hippocampus)	mouse, C57BL/6J	*Actb*, *B2m*, *Gapdh*, *Hmbs*, *Hprt1*, *Oaz1*, *Rps6*, *Tbp*	*Tbp* *Tbp*, *Oaz1*	[55]
Neuroplasticity—methamphetamine	brain (striatum, substantia nigra)	rat, Sprague-Dawley	*18S rRNA*, *B2m*, *Actb*, *Gapdh*, *Hmbs*, *Hprt1*, *Oaz1*, *Rps6*, *Tbp*, *Ubc*	*Actb*,, *Gapdh*, *Hprt1*, *Rps6*	[56]
Neuroplasticity—alcoholism, estrogen	brain, hearth	rat, Sprague-Dawley	*U2*, *U5a*, *U6*, *U87*, *Z39*, *5S rRNA*, *18S rRNA*, *Actb*, *B2m*, *Gadd45af*, *Gapdh*, *Hprt1*, *Tbp*, *Tnks*, *Ubc*	*U87*, *5S rRNA*, *Gapdh*, *U5a*	[57]
autism	brain (prefrontal cortex, hippocampus)	rat, Sprague-Dawley	*Actb*, *Gapdh*, *Hmbs*, *Hprt1*, *Ppia*, *Rpl13a*, *Rps18*, *Tbp*, *Ywhaz*	*Hprt1* *Hmbs*, *Tbp*	[58]
testosterone treatment	brain (hypothalamus), kidney	rat, Sprague-Dawley	*Actb*, *B2m*, *Gapdh*, *Hmbs*, *Hprt1*, *Ppia*	*Hmbs*, *Ppia* *Hmbs*, *Gapdh*	[59]
